# Transcriptomic Analysis of Flower Development in Wintersweet (*Chimonanthus praecox*)

**DOI:** 10.1371/journal.pone.0086976

**Published:** 2014-01-29

**Authors:** Daofeng Liu, Shunzhao Sui, Jing Ma, Zhineng Li, Yulong Guo, Dengpan Luo, Jianfeng Yang, Mingyang Li

**Affiliations:** Chongqing Engineering Research Center for Floriculture, Key Laboratory of Horticulture Science for Southern Mountainous Regions, Ministry of Education, College of Horticulture and Landscape Architecture, Southwest University, Chongqing, China; CSIR Institute of Genomics and Integrative Biology, India

## Abstract

Wintersweet (*Chimonanthus praecox*) is familiar as a garden plant and woody ornamental flower. On account of its unique flowering time and strong fragrance, it has a high ornamental and economic value. Despite a long history of human cultivation, our understanding of wintersweet genetics and molecular biology remains scant, reflecting a lack of basic genomic and transcriptomic data. In this study, we assembled three cDNA libraries, from three successive stages in flower development, designated as the flower bud with displayed petal, open flower and senescing flower stages. Using the Illumina RNA-Seq method, we obtained 21,412,928, 26,950,404, 24,912,954 qualified Illumina reads, respectively, for the three successive stages. The pooled reads from all three libraries were then assembled into 106,995 transcripts, 51,793 of which were annotated in the NCBI non-redundant protein database. Of these annotated sequences, 32,649 and 21,893 transcripts were assigned to gene ontology categories and clusters of orthologous groups, respectively. We could map 15,587 transcripts onto 312 pathways using the Kyoto Encyclopedia of Genes and Genomes pathway database. Based on these transcriptomic data, we obtained a large number of candidate genes that were differentially expressed at the open flower and senescing flower stages. An analysis of differentially expressed genes involved in plant hormone signal transduction pathways indicated that although flower opening and senescence may be independent of the ethylene signaling pathway in wintersweet, salicylic acid may be involved in the regulation of flower senescence. We also succeeded in isolating key genes of floral scent biosynthesis and proposed a biosynthetic pathway for monoterpenes and sesquiterpenes in wintersweet flowers, based on the annotated sequences. This comprehensive transcriptomic analysis presents fundamental information on the genes and pathways which are involved in flower development in wintersweet. And our data provided a useful database for further research of wintersweet and other Calycanthaceae family plants.

## Introduction

The small, evolutionarily ancient Calycanthaceae family comprises four genera, namely *Calycanthus* L., in North America, *Idiospermum* Blake, in Australia, and *Sinocalycanthus* Cheng & S. Y. Chang and *Chimonanthus* L., in China [1]. Wintersweet (*Chimonanthus praecox*), also known as the ‘wax shrub’, is a hardy, fast-growing perennial shrub, native to China; it is dichogamous and diploid (2n = 22) [2]. It is an important deciduous aromatic plant, and it is also one of the most precious epibiotic species dating back to the Tertiary period, being classified as a Class II protected wild plant in China [3]. Wintersweet has over 1000 years’ history of cultivation and, as its name indicates, it blooms particularly in winter, from late November to March in central southern and south-western China. Its unique flowering time and strong fragrance make it one of the most popular ornamental plants in China; it is appreciated as a pot plant and for cut flowers and it has a high ornamental and economic value. It has also been introduced into Korea, Japan, Europe, America, and Australia [2]. Wintersweet flowers are used in traditional Chinese medicinal preparations to treat heatstroke, vomiting, coughs and measles [4]. They have also been recognized as the source of a natural essential oil, which can be used in perfumery, cosmetics and aromatherapy [5], [6]. Recently, these applications of wintersweet have received much attention in New Zealand [7].

There are several traits and properties of wintersweet flowers that are important in a commercial context. These include flower development, senescence, scent biosynthesis and emission, and resistance to biotic and abiotic stresses. Wintersweet blooms especially in winter and has a strong fragrance, and its molecular mechanisms of flower development may therefore be different from those of model species or of plants that bloom in the spring. The molecular and genetic processes that determine some of these flowering characteristics cannot be studied using model species such as *Arabidopsis thaliana*, or at least only to a limited extent. During the past decade, the genes and gene networks associated with important floral traits, including flower scent, color, morphology and senescence, have been identified and functionally characterized in several plants grown for their flowers, such as rose [8], carnation [9], [10], [11], and *Antirrhinum majus* [12], [13]. However, the genomic resources that are available for wintersweet or for other members of the Calycanthaceae family are scant and the transcriptional changes and molecular mechanisms that control these important traits and developmental processes in wintersweet flowers are still far from being elucidated. This hampers gene discovery and seriously hinders the improvement of wintersweet as a commercially important species.

This paucity of genomic information is indicated by the fact that although a normalized cDNA library has been constructed from flowers of wintersweet and 479 unigenes have been assembled [14], nevertheless as of late October 2013 only 867 expressed sequence tags (ESTs) for *Chimonanthus praecox* were available on the NCBI website (http://www.ncbi.nlm.nih.gov/). Because gene identification and characterization in *Chimonanthus praecox* remain so limited, the large-scale discovery and characterization of functional genes via genome sequencing or exploration of the transcriptome is essential. The high-throughput capacity of the latest generation of RNA sequencing (RNA-Seq) technology provides a unique opportunity for genomic exploration and gene discovery in non-model plant species for which there are no reference genome sequence data [15], [16]. The results of RNA-Seq also show high levels of reproducibility, for both technical and biological replicates [17], [18]. RNA-Seq generates absolute rather than relative gene expression measurements, providing greater insight and accuracy than microarrays [19], [20].

In this study, we used the Illumina RNA-Seq method to analyze the transcriptome of wintersweet flowers based on three cDNA libraries from different stages of flower development. A total of 106,995 transcripts were identified, and the sequences were annotated against the NR database using BLASTX. By using Illumina’s digital gene expression platform, we investigated differential gene expression in open and in senescing flowers, and analyzed the selective expression of Kyoto Encyclopedia of Genes and Genomes (KEGG) pathways. In particular, we identified genes of plant hormone signal transduction pathways that were differentially expressed during flower development, and also key genes of floral scent biosynthesis. To our knowledge, this is the first comprehensive transcriptomic study to identify genes and pathways that are differentially expressed during flower opening and senescence in wintersweet. It provides an important new bioinformatics resource for the further identification of genes and gene function involved in flower development in this species.

## Results

### Illumina Sequencing and the Assembly of Sequence Reads

**Figure 1:**
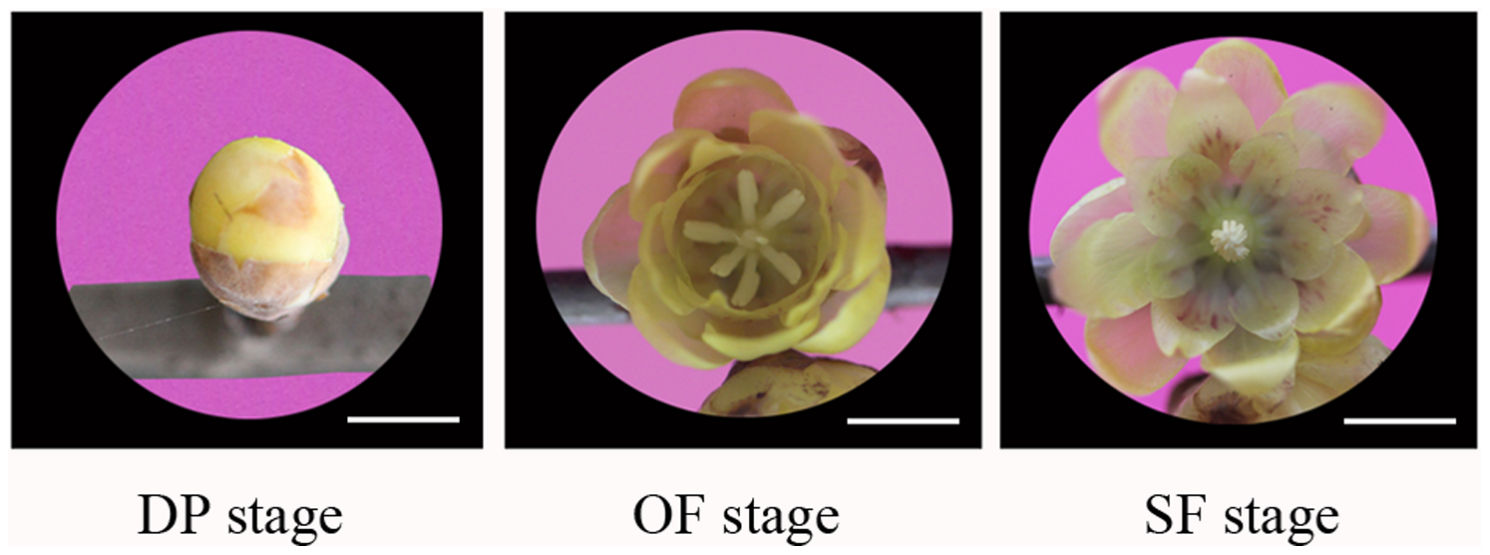
Flower development stages in wintersweet. Pictures of wintersweet flowers: at flower bud stage with displayed petal (DP), at open flower stage (OF); and at senescing flower stage (SF). Scale bar = 1 cm.

**Figure 2:**
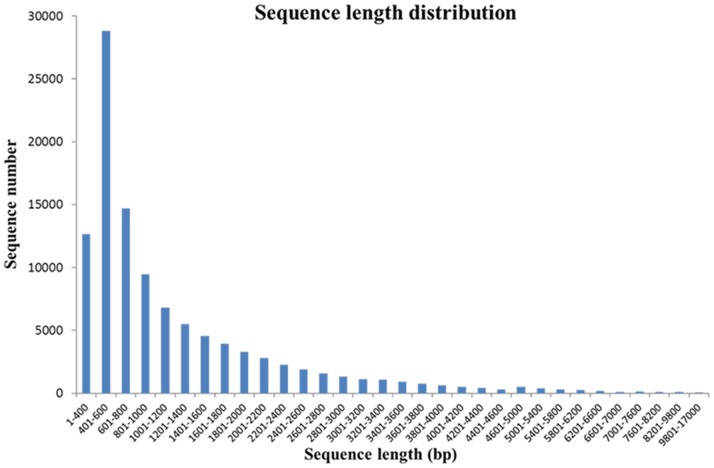
Sequence-length distribution of transcripts assembled from Illumina reads. All the Illumina reads for each flower development stage (see Fig. 1) were combined together (see text) and gave rise to 106,995 transcripts. The horizontal and vertical axes show the size and the number of transcripts, respectively.

In this study, we divided wintersweet flower development into three essentially distinct stages: i) bud stage, with the flower buds enlarged and turned yellow and with a displayed petal (DP); ii) open flower stage (OF), with the flowers fully opened and emitting a strong fragrance; and iii) senescing flower stage (SF), with withering petals and loss of fragrance (Figure 1). Three individual flowers were used to prepare one pooled RNA sample for each of the three developmental stages (DP, OF and SF). Three cDNA libraries were then constructed and subjected to Illumina deep sequencing. We obtained 21,412,928, 26,950,404, and 24,912,954 qualified Illumina reads, respectively, for the DP, OF and SF stages, giving rise to 2,113,119,501, 2,653,944,208 and 2,451,552,573 bp total residues, respectively (Table 1). After eliminating primer and adapter sequences and filtering out the low-quality reads, we pooled together all the high-quality Illumina reads from the three different developmental-stage libraries. Using Trinity software, we then combined all the reads to form a transcriptome database for wintersweet [21]. We identified 106,995 transcripts, with total residues of 127,396,344 bp. The average length of each transcript was 1,190 bp, the shortest sequence being 351 bp and the longest being 16,998 bp (Table 1). The sequence length distribution of transcripts is indicated in Figure 2. Most of the transcripts (40.6%) were 401∼800 bp in length (Table S1).

**Table 1:** Summary of Illumina transcriptome sequencing for wintersweet flower.

	DP	OF	SF
**Reads**	21,412,928	26,950,404	24,912,954
**Base number (bp)**	2,113,119,501	2,653,944,208	2,451,552,573
**Total residues (bp)**		127,396,344	
**Number of transcripts (n)**		106,995	
**Average length (bp)**		1190.68	
**Largest transcripts (bp)**		16,998	
**Smallest transcripts (bp)**		351	

### Sequence Annotation

**Figure 3:**
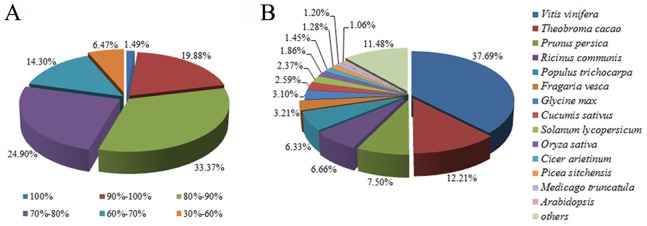
Results summary for sequence-homology search against NCBI NR database. (A) Similarity distribution of the closest BLASTX matches for each sequence. (B) A species-based distribution of BLASTX matches for sequences. We used all the plant proteins in the NCBI NR database in performing the homology search and for each sequence we selected the closest match for analysis.

For annotation, the sequences were searched against the NCBI non-redundant (NR) database using BlastX, setting a cut-off E-value of 10^−5^. A total of 51,793 (48.4%) sequences showed significant similarity to known proteins in the NR database. The similarity distribution for all the search results showed that 54.7% of the matches were of high similarity, *i.e.* ranging from 80% to 100% similarity as reported in the BlastX results, whilst 39.2% of the matches were of similarity ranging from 60% to 80% (Figure 3A). In a further analysis of the matching sequences, we found that 37.7% of the sequences showed closest matches with sequences from *Vitis vinifera*. The next-closest matches were with sequences from *Theobroma cacao* and *Prunus persica*; 12.2% of the sequences showed closest matches with sequences from *Theobroma cacao*, whilst 7.5% of the sequences showed closest matches with sequences from *Prunus persica* (Figure 3B).

### GO, COG and KEGG Classification

**Figure 4:**
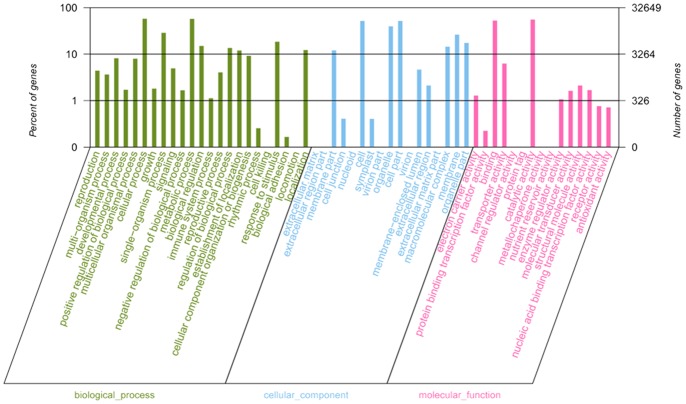
Gene ontology (GO) assignments for the flower transcriptome of wintersweet. Results are summarized under three main GO categories: biological process, cellular component and molecular function. The left y-axis indicates the percentage of a specific category of genes in each main category. The right y-axis indicates the number of genes in the same category.

We added gene ontology (GO) terms using Blast2GO [22], which annotates high-score BLAST matches found to sequences in the NCBI NR proteins database. Genes of wintersweet were classified into three main GO categories and 57 sub-categories. Of the 106,995 assembled transcripts, 32,649 were successfully annotated by GO assignments, and some of them belonged to one or more of the three categories (Figure 4). Amongst the annotated sequences, biological process categories included cellular process (18,805; 57.6%), metabolic process (18,692; 57.3%), single-organism process (9,356; 28.7%), response to stimulus (6,010; 18.4%), biological regulation (4,880; 15.0%), regulation of biological process (4,417; 13.5%), and other categories (23,906; 73.2%). Furthermore, cellular component categories included cell part (16,837; 51.6%), cell (16,837; 51.6%), organelle (12,905; 39.5%), membrane (8,561; 26.2%), organelle part (5,671; 17.4%), macromolecular complex (4,709; 14.4%), membrane part (3,919; 12.0%), and other categories (2,551; 7.8%). In addition, molecular function categories included catalytic activity (18,042; 55.3%), binding (17,154; 52.5%), transporter activity (2,044; 6.3%), structural molecule activity (690; 2.1%), nucleic acid binding transcription factor activity (549, 1.7%), molecular transducer activity (530; 1.6%), and other categories (1,355; 4.2%) (Table S2).

**Figure 5:**
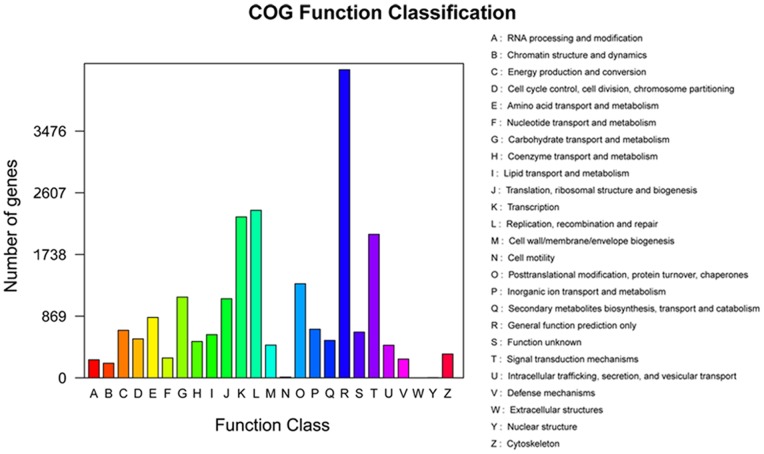
COG functional classification for the flower transcriptome of wintersweet. From a total of 106,995 *de novo* assembled transcripts, 21,893 transcripts with significant homologies in the COG database (E-value ≤1.0 E-5) were classified into 25 COG categories.

In a further analysis, the annotated sequences were subjected to a search against the Clusters of Orthologous Group (COG) database, for functional prediction and classification. Based on sequence homology, 21,893 unique sequences were assigned a COG functional classification. These sequences were classified into 25 COG categories, denoting involvement in cellular process, signal transduction, metabolism and other processes (Figure 5). The most common category was the non-specific category of ‘general function prediction only’ (4,341; 19.8%), followed by replication, recombination and repair (2,360; 10.8%), transcription (2,267; 10.4%), signal transduction mechanisms (2,022; 9.2%), posttranslational modification, protein turnover and chaperones (1,326; 6.1%), and carbohydrate transport and metabolism (1,138; 5.2%). Amongst other categories represented (Table S3) were translation, ribosomal structure and biogenesis (1,115; 5.1%) and amino acid transport and metabolism (851; 3.9%). The categories of cell motility (11; 0.05%) and nuclear structure (2; 0.01%) were the least represented.

In order to identify biochemical pathways, we mapped the annotated sequences onto the KEGG database, which is an alternative approach to the categorization of gene function and which places emphasis on biochemical pathways. In total, 15,587 transcripts were assigned to 312 KEGG pathways. A summary of the findings is presented in Table S4. The largest number of sequences were those associated with metabolic pathways (4,293; 27.5%), followed by sequences that were involved in the biosynthesis of secondary metabolites (2,049; 13.2%), in microbial metabolism in diverse environments (831; 5.3%), in the biosynthesis of amino acids (495; 3.2%), in plant hormone signal transduction (467; 3.0%), and in a number of other pathways. In particular, several important pathways of secondary metabolite biosynthesis, including the phenylpropanoid, flavonoid and carotenoid biosynthetic pathways, and several signaling pathways, including the p53, mitogen-activated protein kinase (MAPK) signaling pathway, were represented. These pathway assignments provide valuable information for the investigation of specific biochemical and development processes.

### Differentially Expressed Genes (DEGs) during Flower Development

**Figure 6:**
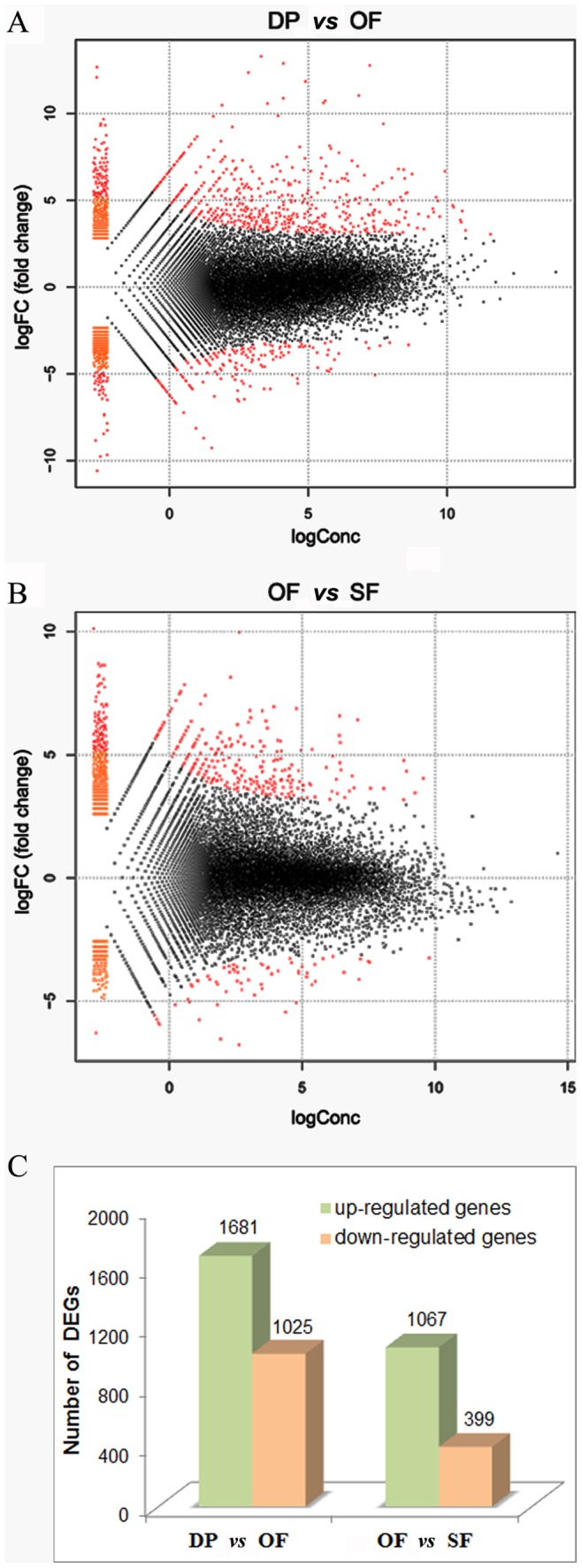
Differentially expressed genes (DEGs) in the flower development-stage comparisons DP vs OF and OF vs SF. (A) Whole-study overview of log-fold changes in gene expression in DP *vs* OF. (B) Whole-study overview of log-fold changes in gene expression in OF *vs* SF. The x-axis indicates the absolute expression levels (LogConc). The y-axis indicates the log-fold changes between the two samples. Genes for which differential expression is significant are shown as red dots (Log_2_FC≥2 or ≤−2; FDR≤0.05). (C) The number of up- or down-regulated genes in DP *vs* OF and OF *vs* SF. DP *vs* OF refers to the comparison between the bud stage showing a displayed petal (DP) and the open flower stage (OF). OF *vs* SF refers to the comparison between the open flower stage (OF) and the senescing flower stage (SF).

To study gene expression during flower development, a genome-wide expression analysis was carried out for each of the DP, OF and SF flower developmental stages (Figure 1). We mapped the Illumina reads from each stage onto our assembled transcriptome database. The expression of each gene was calculated using the numbers of reads mapping onto its transcripts. Comparisons of gene expression between DP and OF (DP *vs* OF) and between OF and SF (OF *vs* SF) showed that 2,706 and 1,466 genes were differentially expressed in DP *vs* OF and in OF *vs* SF, respectively (Log_2_ fold changes: ≥2 or ≤−2; FDR ≤0.05). An overall view of the expression pattern for DP *vs* OF is depicted in Figure 6A. This shows the expression changes for 2,706 differentially expressed genes (DEGs), ranging from a 14-fold to a -12-fold change in expression. A corresponding view for OF *vs* SF is depicted in Figure 6B, which shows the changes for 1,466 DEGs, ranging from a 12-fold to an -8-fold change in expression. Figure 6C illustrates that for DP *vs* OF there were 1,681 up-regulated genes and 1,025 down-regulated genes, whilst in the case of OF *vs* SF, there were 1,067 and 399, respectively.

To investigate further, the 20 most up-regulated and the 20 most down-regulated genes were each selected from both the DP *vs* OF and the OF *vs* SF comparisons. Some of these genes showed matches to sequences in the NCBI NR database, such as cytochrome P450, α-terpineol synthase, lipid-transfer protein, cysteine protease, and GDSL esterase/lipase (Tables S5; S6). For example, we found that a cytochrome P450 gene was the most up-regulated gene (13-fold) at the OF stage but was down-regulated substantially at the SF stage (Table S5). Two cysteine protease genes were down-regulated with −10.9 and −8.9 fold changes respectively at the OF stage, and showed no expression at all at the SF stage (Table S5). Finally, we found that a GDSL esterase/lipase gene was induced specifically and strongly (10-fold change in expression) at the SF stage (Table S6).

### Pathway Enrichment Analysis of DEGs

**Figure 7:**
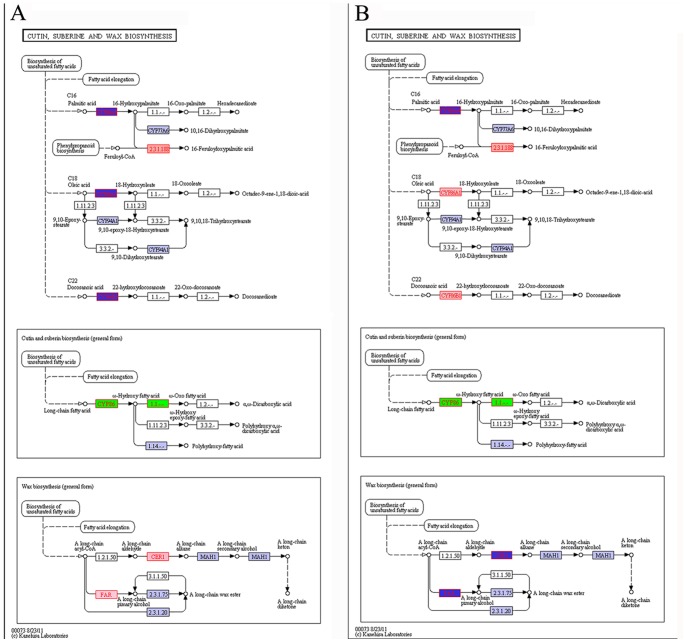
Regulatory changes in the pathway of cutin, suberin and wax biosynthesis during flower development. (A) Gene expression changes between DP and OF stages. (B) Gene expression changes between OF and SF stages. Green boxes, genes identified in our data; light blue boxes, genes involved in the pathway present in the KEGG database but undetectable in our data; pink boxes, up-regulated genes; dark blue boxes, down-regulated genes.

The KEGG Orthology Based Annotation System (KOBAS) [23] was used for the further identification of biosynthetic and other pathways and to explore in greater depth the functions of DEGs. Pathways that were found to be significantly up-regulated (corrected P-value ≤0.05) in both DP *vs* OF and OF *vs* SF were involved in cutin, suberin and wax biosynthesis; cyanoamino acid metabolism; pentose and glucuronate interconversions; phenylalanine metabolism; phenylpropanoid biosynthesis; plant hormone signal transduction; and starch and sucrose metabolism (Table 2). Since wintersweet, otherwise known as ‘wax shrub’, blooms in winter and displays a strong resistance to biotic and abiotic stress, we focused upon the pathway of cutin, suberin and wax biosynthesis, and obtained matches to key enzymes of cutin, suberin and wax biosynthesis, such as CYP86A1, CYP86A4, CYP86B1, ECERIFERUM (CER), and alcohol-forming fatty acyl-coenzyme A reductases (FARs). The expression of these genes was found to be differentially regulated during flower development in wintersweet. In addition, the *CER1* and *FAR* genes, which are related to wax biosynthesis, were found to be up-regulated at the OF stage and down-regulated at the SF stage (Figure 7).

**Table 2:** Statistically enriched pathways identified using KOBAS database in differentially expressed transcripts during flower development.

			DP *vs* OF			OF *vs* SF		
Pathway	Pathway ID	Bn	Nt	P-Value	Corrected P-Value	Nt	P-Value	Corrected P-Value
**beta-Alanine metabolism**	ko00410	114	27	0.000875	0.021922			
**Biosynthesis of unsaturated fatty acids**	ko01040	76				19	6.01E-06	0.000441
**Cutin, suberine and wax biosynthesis**	ko00073	61	28	3.07E-11	1.16E-08	19	6.96E-08	8.52E-06
**Cyanoamino acid metabolism**	ko00460	104	30	2.32E-06	0.000145	22	1.02E-05	0.000626
**Flavone and flavonol biosynthesis**	ko00944	11	6	0.001135	0.026674			
**Flavonoid biosynthesis**	ko00941	80	27	7.28E-09	9.12E-07			
**Galactose metabolism**	ko00052	175				26	0.001580	0.048309
**Histidine metabolism**	ko00340	57	17	0.000442	0.015111			
**Limonene and pinene degradation**	ko00903	36	13	0.000766	0.020561			
**Methane metabolism**	ko00680	213				39	1.53E-07	1.40E-05
**Nicotinate and nicotinamide metabolism**	ko00760	52	16	0.000333	0.012529			
**One carbon pool by folate**	ko00670	49	15	0.000672	0.019441			
**Pentose and glucuronate interconversions**	ko00040	124	43	9.51E-11	1.79E-08	20	0.001866	0.048919
**Peroxisome**	ko04146	238	48	0.000148	0.006188			
**Phenylalanine metabolism**	ko00360	111	25	0.001618	0.035787	22	4.44E-05	0.002327
**Phenylpropanoid biosynthesis**	ko00940	185	51	1.46E-08	1.37E-06	42	3.37E-10	1.24E-07
**Plant hormone signal transduction**	ko04075	467	92	3.65E-06	0.000196	72	4.62E-08	8.47E-06
**Porphyrin and chlorophyll metabolism**	ko00860	134				23	0.000525	0.024080
**Starch and sucrose metabolism**	ko00500	412	86	6.34E-08	4.77E-06	50	0.000638	0.025562
**Stilbenoid, diarylheptanoid and gingerol biosynthesis**	ko00945	21	10	0.000107	0.005008			

Bn (Back ground number), indicates the total number of transcripts present in each pathway. Nt (Number of transcripts), indicates the number of differentially expressed transcripts in each pathway.

We also focused upon plant hormone signal transduction pathways, apparently for the first time in wintersweet, almost all the key genes could be identified from our data (Figure S1). We identified 92 and 72 transcripts associated with plant hormone signal transduction pathways that were differentially expressed in DP *vs* OF and OF *vs* SF, respectively (Table 2). The DEGs were identified as phytohormone receptor or phytohormone-responsive genes known to be induced by various phytohormone signaling pathways, including those for auxins, cytokinins (CK), abscisic acid (ABA), ethylene (ET), brassinosteroids (BR), jasmonic acid (JA) and salicylic acid (SA), as indicated in Table 3. The early auxin-responsive genes (*i.e.* those associated with the early phase of a response to auxins) have been grouped broadly into three major classes: the auxin/indole-3-acetic acid (Aux/IAA), the GH3, and the small auxin-up RNA (SAUR) gene families. Our data showed that the *Aux/IAA*, *GH3* and *SAUR* genes were expressed differentially during wintersweet flower opening and senescence. Moreover, a number of JA-responsive genes, including *JAR* (JAR Jasmonate Resistant 1), *JAZ* (Jasmonates ZIM-domain protein) and *MYC2*, were all induced significantly at the OF stage, whereas the salicylic acid-responsive genes, *TGA* and *PR1*, were induced specifically and strongly at the SF stage (Table 3).

**Table 3:** Differentially expressed genes related to plant hormone signal transduction pathway during flower development.

			DP *vs* OF		OF *vs* SF	
Gene ID	Putative function	Nearest match	log_2_ FC	FDR	log_2_ FC	FDR
	**Auxin signal transduction**					
Cp13004_c0	auxin influx carrier protein (AUX1, LAX)	ABN81349.1	−2.51	0.013927	2.69	0.010811
Cp1471_c0	auxin influx carrier protein (AUX1, LAX)	CCF23026.1	4.25	2.69E-06		
Cp1637_c0	Aux/IAA	EMJ16790.1			2.28	0.035763
Cp6390_c0	Aux/IAA	XP_002304179.1	5.65	8.28E-09	−3.89	3.68E-05
Cp15215_c0	auxin response factor (ARF)	BAD19061.1	3.97	0.012847		
Cp17433_c0	auxin response factor (ARF)	XP_002326300.1	2.92	0.017690		
Cp39410_c0	auxin response factor (ARF)	XP_003634382.1			2.66	0.016236
Cp17028_c0	Auxin-responsive GH3 family protein (GH3)	AFC36444.1			3.06	0.006564
Cp19730_c0	Auxin-responsive GH3 family protein (GH3)	EOY12664.1	−2.59	0.020622		
Cp7344_c0	Auxin-responsive GH3 family protein (GH3)	AAD32141.1	5.14	1.01E-06	2.24	0.025821
Cp10520_c1	SAUR family protein	EOX96266.1			3.33	0.000578
Cp13060_c0	SAUR family protein	XP_002266248.1	3.78	0.000358		
Cp13833_c0	SAUR family protein	EOX96266.1			4.69	1.08E-05
Cp15265_c0	SAUR family protein	XP_002272614.1	3.10	0.005876		
Cp17085_c0	SAUR family protein	XP_002265932.1	6.72	0.001457		
Cp21781_c0	SAUR family protein	XP_002327252.1	−4.69	0.001700		
Cp24119_c0	SAUR family protein	XP_002968676.1			−4.21	0.017503
Cp3195_c0	SAUR family protein	EOX99720.1			3.18	0.001959
Cp53091_c0	SAUR family protein	CAN62213.1	6.14	0.010751		
	**CK signal transduction**					
Cp38923_c0	histidine phosphotransfer protein (AHP)	XP_002298243.1			4.16	0.020819
Cp21971_c0	type A response regulator (ARR-A)	XP_002302230.1	−8.09	4.17E-06		
Cp4567_c0	type A response regulator (ARR-A)	XP_002267404.1	2.33	0.012744		
	**ABA signal transduction**					
Cp13360_c0	abscisic acid receptor PYL-like protein (PYL)	XP_002516457.1	7.15	3.62E-08	−3.33	0.001215
Cp7252_c0	abscisic acid receptor PYL-like protein (PYL)	XP_002264158.1	9.81	5.39E-10		
Cp22532_c0	type 2C protein phosphatases (PP2C)	XP_002318387.1			5.65	2.51E-06
Cp9742_c0	type 2C protein phosphatases (PP2C)	XP_004291511.1			3.37	0.000739
Cp2918_c0	SNF1-related kinase 2 (SnRK2)	XP_002262726.1	2.67	0.003540		
Cp7416_c0	ABRE binding factors (ABF)	XP_002326942.1			2.59	0.014829
	**Ethylene signal transduction**					
Cp14969_c0	EIN3 binding F-box (EBF1/2)	AEK81539.1	6.49	1.98E-06		
	**Brassinosteroid signal transduction**					
Cp2974_c0	BRI1 KINASE INHIBITOR 1 (BKI1)	CAN78408.1	4.46	1.10E-06		
Cp11265_c0	CYCD3	BAA76478.1	−2.66	0.007958		
	**JA signal transduction**					
Cp930_c0	Jasmonate Resistant 1 (JAR1)	EOX95769.1	3.65	5.34E-05	−2.38	0.024910
Cp1074_c0	jasmonates ZIM-domain protein (JAZ)	XP_002529200.1	3.17	0.000610		
Cp12614_c0	jasmonates ZIM-domain protein (JAZ)	XP_002304118.1	3.95	0.000207		
Cp1550_c4	jasmonates ZIM-domain protein (JAZ)	XP_002534018.1	2.18	0.033780		
Cp2223_c0	jasmonates ZIM-domain protein (JAZ)	ADI39634.1	3.07	0.001190		
Cp2936_c0	jasmonates ZIM-domain protein (JAZ)	XP_002284855.1	3.75	4.30E-05	−2.59	0.007908
Cp12298_c0	MYC2	EOY23994.1	2.25	0.018227		
	**SA signal transduction**					
Cp22279_c0	BZIP transcription factor family protein (TGA)	XP_003554196.1	−4.21	0.000286		
Cp47428_c0	BZIP transcription factor family protein (TGA)	EOY09442.1			6.35	0.015379
Cp9043_c0	BZIP transcription factor family protein (TGA)	EOX92234.1			2.64	0.012478
Cp3374_c0	pathogenesis-related protein 1 (PR1)	BAF95881.1			8.03	6.50E-12

Differentially expressed genes with Log_2_ fold changes ≥2 or ≤−2, and FDR ≤0.05 were included.

The genes involved in the pathway are color-coded: green boxes, genes identified in our data; and light blue boxes, genes involved in the pathway present in KEGG database but undetectable in our data.

### Genes of Floral Scent Biosynthesis

**Figure 8:**
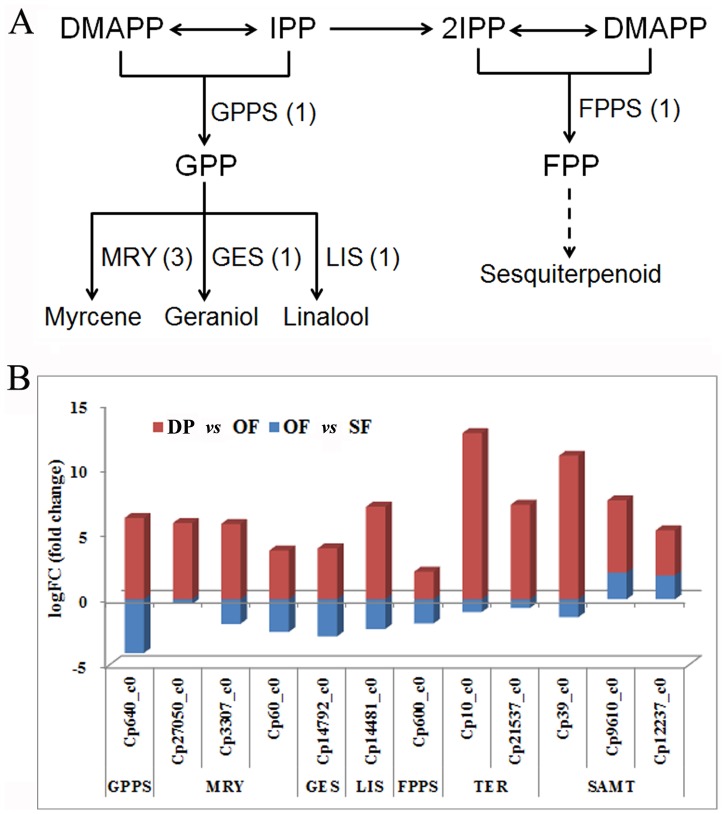
Proposed biosynthetic pathway for monoterpenes and sesquiterpenes in wintersweet. (A) Proposed biosynthetic pathway for monoterpenes and sesquiterpenes. (B) Changes in expression (as –fold) of key genes of floral scent biosynthesis during flower development. DMAPP: dimethylallyl diphosphate; IPP: isopentenyl diphosphate; GPPS: geranyl diphosphate synthase; FPPS: farnesyl pyrophosphate synthase; MRY: myrcene synthase; GES: geraniol synthase; LIS: S-linalool synthase; TER: α-terpineol synthase; SAMT: S-adenosyl-L-methionine: salicylic acid carboxyl methyltransferase. Each enzyme name is followed in parentheses by the number of gene homologues encoding this enzyme. Solid lines indicate direct biochemical reactions, and broken lines indicate indirect reactions.

Wintersweet flowers develop a strong and specific fragrance during flower opening. In this study, we obtained from our transcriptomic data the key biosynthetic genes of floral scent production, including GPPS (geranyl diphosphate synthase), MRY (myrcene synthase), GES (geraniol synthase), LIS (S-linalool synthase), FPPS (farnesyl pyrophosphate synthase), TER (α-terpineol synthase), and SAMT (S-adenosyl-L-methionine: salicylic acid carboxyl methyltransferase). These included three homologous genes of *SAMT* and *MRY* and two homologous genes of *TER*. Based on the annotated sequences, we have therefore proposed a biosynthetic pathway for the formation of monoterpenes and sesquiterpenes in wintersweet (Figure 8A).

The expression of genes of floral scent biosynthesis was induced at the OF stage (Figure 8B). The expression of the *LIS* gene, which is responsible for α-linalool biosynthesis, was increased 7-fold at the OF stage; and it has been reported that α-linalool accounts for 36% of the total quantity of volatile compounds detected in wintersweet flowers [6]. Similarly, methyl salicylate has also been demonstrated to be one of the major volatile compounds in wintersweet flowers [6]; and the three *SAMT* gene homologues, responsible for the conversion of salicylic acid to methyl salicylate, were all induced significantly to varying extents at the OF stage.

### Real-time Quantitative PCR Validation of RNA-Seq Results

**Figure 9:**
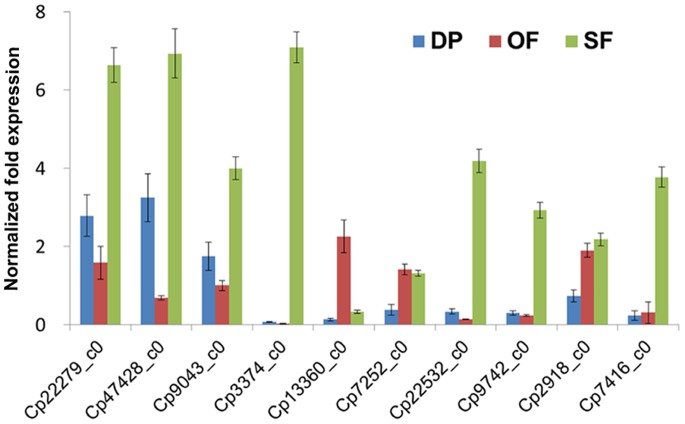
Real-time qPCR validation of ten genes involved in plant hormone signal transduction pathways. Data were normalized against a reference of wintersweet actin and tubulin genes. All quantitative PCRs for each gene used three biological replicates, with three technical replicates per experiment; the error bars indicate SD.

To validate the findings from the sequencing data, an appropriate alternative methodology was chosen. Ten DEGs involved in plant hormone signal pathways were selected for validation using Real-time qPCR, with gene-specific primers designed using Primer Primer software (version 5.0), as shown in Table S7. The expression pattern of these genes at each of the three flower developmental stages is shown in Figure 9. Expression patterns determined by Real-time qPCR were consistent with those obtained by RNA-Seq, confirming the accuracy of the RNA-Seq results reported in this study.

## Discussion

### Illumina Sequencing and Sequence Annotation

Wintersweet (*Chimonanthus praecox*) is mainly a garden plant and a traditional cut flower in China. However, little genomic information is available for this species. In this study, we have generated large amounts of cDNA sequence data, comprising long sequences of good quality, which will facilitate subsequent and more detailed studies. The availability of transcriptomic data for wintersweet will meet the basic requirements needed to undertake a thorough molecular-genetic and biochemical characterization of this species and its relatives.

For gene annotation, the sequences we had determined were searched against the NCBI NR database. Because of the limited genetic information available, no matches were obtained for as many as 55,202 (51.6%) of the sequences. Some of these sequences may represent novel genes in wintersweet, suggesting that flower development in wintersweet might involve some unique processes and pathways. Interestingly, the annotated sequences of wintersweet showed a higher homology to proteins of *Vitis vinifera* (Figure 3), which may reflect the evolutionary relationship between *Chimonanthus praecox* and *Vitis vinifera*. *Chimonanthus praecox* (Calycanthaceae) belongs to the order Laurales and to the clade magnoliids, which arose in evolution soon after the divergence of the eudicots. *Vitis vinifera* (Vitaceae) belongs to the clade rosids, which lies at the root of the eudicots, and which is closer phylogenetically to the clade magnoliids than to the order Malvales (e.g. *Theobroma cacao*), or to the order Rosales (e.g. *Prunus persica*).

In spite of the large proportion of sequences that showed no matches, a large number of transcripts were nevertheless assigned to a wide range of GO and COG classifications, which indicated that our transcriptome data represented a broad diversity of transcripts in wintersweet. KEGG predictions identified many genes associated with secondary metabolites, including genes of phenylpropanoid biosynthesis, flavonoid biosynthesis, phenylalanine metabolism and carotenoid biosynthesis; in addition, we identified genes related to three alkaloid biosynthesis pathways (Table S4). Alkaloids as a class often exhibit antibacterial and immunostimulatory properties, and this would appear to be consistent with the medicinal and health-promoting properties that are associated with wintersweet [24].

### DEGs during Flower Development

Although the analysis of DEGs during flower opening and senescence has been performed using microarrays in *Iris* [25], *Alstroemeria* [26], and *Rosa* [27], nevertheless the availability of data relating to developmental expression profiles and comparing the transcriptomes of flowers or flower parts at different stages remains generally limited. The approach we used here identified >4000 DEGs, at a cut-off of 5% FDR, that showed differential expression patterns in the pairings, DP *vs* OF and OF *vs* SF. Further insights were obtained by examining the changes of gene expression for sets of related genes. Thus, amongst the 20 most up-regulated DEGs, we found one cytochrome P450 gene that was up-regulated significantly at the OF stage and down-regulated at the SF stage (Table S5); this is in apparent contrast to a report of increased expression of cytochrome P450 genes in senescing petals of *Alstroemeria* [26]. Cytochrome P450 genes are found in all kingdoms and show extraordinary diversity in their chemical reactions [28]; they are one of the largest gene families in plants, although the functions of cytochrome P450 genes in flowers are as yet unknown.

In our study, two cysteine protease genes were down-regulated significantly at the OF stage, and showed no expression at all at the SF stage (Table S5). This appears to contrast with a range of observations showing an up-regulation of cysteine proteases during flower senescence, in both ethylene-sensitive and ethylene-insensitive flowers, such as *Dianthus caryophyllus* (carnation) [29], *Hemerocallis spp*. (daylily) [30], *Alstroemeria peruviana* [31], *Sandersonia aurantiaca* [32], *Narcissus pseudonarcissus* (daffodil) [33], and *Gladiolus grandiflora* [34]. This suggests that there may be a different function, or regulation mechanism, for cysteine protease genes in wintersweet, compared to other species.

### Genes Related to Plant Hormone Signaling Pathways

Plant hormones are involved in many different processes throughout the life of a plant, including growth, development and senescence [35]. Several plant hormones such as ET, ABA, GA have been reported to play important roles in flower opening and in senescence processes. The specific regulation of flower development is a complex process and is governed both by the endogenous levels of the hormones and by the sensitivity of the hormone response mechanisms. The expression of numerous genes has been found to be altered through the action of plant hormone signaling pathways, as well as by the diversity of interactions amongst plant hormones themselves [36].

Ethylene is one of the most important plant hormones, and it plays a major role in many flowers, such as rose, carnation and orchids, in regulating flower opening, senescence and abscission. A burst of endogenously produced ethylene in such flowers initiates senescence and coordinates the expression of genes required for the process [37]. In wintersweet, however, previous research indicated that no obvious production of ethylene could be detected from the flowers during flower development [38]. In the work we report here, we obtained families of genes encoding ethylene receptors (ETR1, ETR2, EIN4, ERS1 and ERS2), EIN3/EILs (EIN3-like proteins), and the ethylene response factor (ERF); in no cases, however, did we find that the expression of these genes was induced significantly during flower opening and senescence. These findings therefore indicated that flower opening and senescence in wintersweet may not be dependent upon an ethylene-mediated signal transduction process triggered by the endogenous production of small amounts of ethylene. On the other hand, it has been reported that ethephon treatment of wintersweet cut flowers can accelerate the process of flower opening, indicating that wintersweet flowers are sensitive to exogenous ethylene treatment [39].

Salicylic acid (SA) or its derivatives function in a diversity of plant processes, such as seed germination, respiration, stomatal responses, senescence and responses to abiotic stress [40]. Many of the components of the SA signaling pathway, including those of signal perception, have not yet been revealed [41]. However, it is known that SA or its signaling pathway is associated with the activation of diverse groups of defense-related genes, including those encoding pathogenesis-related (PR) proteins [42]. Moreover, the non-expresser of PR genes 1 (NPR1) and transcription factors such as TGACG-motif binding factors (TGAs) have been identified as key components of the SA response [43]. In our present study, the *TGA* and *PR1* genes were found to be up-regulated specifically and significantly at the SF stage (Table 3), suggesting that SA may be involved in the regulation of wintersweet flower senescence. The reported involvement of SA in defense responses and in reactions to abiotic stresses in plants may also be relevant, since wintersweet blooms in winter when temperatures are low.

In addition, we found that a range of auxin-related, ABA-related and JA signaling pathway-related genes isolated in our study, such as *Aux/IAA*, *ARF*, *GH3*, *SAUR,JAR1*, *JAZ*, *MYC2*, *SnRK2* and *ABF*, were differentially regulated during flower opening and senescence in wintersweet.

Taken together, these findings suggest that flower development in wintersweet is subject to complex regulation by multiple hormones. The regulatory mechanisms by which phytohormones are involved in wintersweet flower opening and senescence are still far from elucidated. The identification in this study of genes related to plant hormone signaling pathways is therefore an essential step in understanding fully the hormonal regulation of flowering and flower development in wintersweet and other winter-flowering species.

### Floral Scent Biosynthesis and its Genetic Characterization

Floral scents are unique and distinct for each plant. A distinctive floral scent plays an important role in the reproductive processes of many plants and also enhances the aesthetic properties and economic value of ornamental plants and cut flowers. Flower scents vary between plant species on account of their characteristic profiles of volatile compounds of low molecular mass, such as monoterpenes, seisquiterpenes, benzenoids, phenylpropanoids, and fatty acid derivatives [44]. Many of these volatile compounds have already been identified in a number of flower species and in addition many of the biosynthetic enzymes involved have been identified and functionally characterized [45]. Some pathways have been characterized at the molecular-genetic level, primarily in model systems such as *Clarkia breweri* [46]–[50], *Petunia* [51], rose [52], [53], and *Antirrhinum* [50].

In a previous study, more than 30 volatile compounds had been detected in wintersweet flowers; these consisted almost exclusively of volatile terpenoids (monoterpenes and sesquiterpenes) and benzenoids [5], [6]. There was little molecular-genetic characterization of the biosynthesis of these compounds in wintersweet, however; and to the best of our knowledge, only the *FPPS* (farnesyl pyrophosphate synthase) gene and a homologue of the *SAMT*(S-adenosyl-L-methionine: salicylic acid carboxyl methyltransferase) gene had been cloned [54], [55].

In this study, we isolated additional key floral scent-synthesizing genes from wintersweet, based on sequence annotations and analyses of changes in gene expression during flower development. The expression of the *FPPS* gene was found to be induced significantly during flower opening, consistent with previous findings, and was correlated with the emission of sesquiterpenoids [54]. Terpineol synthases (TERs) have been isolated from plant species representing six genera: *Magnolia grandiflora* [56], *Pinus taeda* [57], *Santalum album* [58], *Vitis vinifera* [59], *Zea mays* [60] and *Nicotiana* species [61]. The TER enzyme of *M. grandiflora* produces the single product, α-terpineol, whilst the TER enzymes of the other species produce additional compounds, such as limonene, β-myrcene, α-pinene, and β-pinene [61]. In wintersweet flowers, both α-terpineol and α-pinene have been detected [4], [5].

The identification of genes of floral scent biosynthesis will facilitate future molecular-genetic and physiological studies of the regulation of floral scent production during flower development in wintersweet. It may also open new opportunities for the metabolic engineering of floral volatiles in plants [62].

## Conclusions

This work presents the first *de novo* transcriptome sequencing analysis of wintersweet flower development using the Illumina RNA-Seq method. A total of 10,699 transcripts were assembled and 51,793 sequences were annotated. Our work provides an excellent platform for future genetic and functional genomics research in wintersweet and also provides an invaluable resource for genomics studies in other members of the Calycanthaceae family. Using Illumina sequencing-based gene analysis for the identification of DEGs, we identified a large number of candidate genes that were regulated either at the OF stage or at the SF stage, or at both, and we were able to identify a number of regulated pathways. In particular, we identified DEGs that were involved in plant hormone signal transduction pathways during flower development. The results indicated that flower opening and senescence may not be dependent upon the ethylene signal transduction pathway in wintersweet; in contrast, however, SA may be involved in the regulation of flower senescence. Finally, we obtained a number of key genes of floral scent biosynthesis and on this basis we have proposed a biosynthetic pathway for monoterpenes and sesquiterpenes in wintersweet flower based on annotated sequences. In summary, this comprehensive transcriptomic analysis has provided essential information on the genes and pathways which are involved in flower development in wintersweet. It will be fundamental to the development of wintersweet cultivars of improved horticultural or ornamental quality.

## Materials and Methods

### Plant Materials

Flowers of wintersweet (*Chimonanthus praecox* (L.) Link) were collected from the nursery at Southwest University, Chongqing, China. Flowers at flower bud stage with displayed petal (DP), open flower stage (OF) and senescing flower stage (SF) were dissected from the plants and immediately frozen and stored in liquid nitrogen.

### RNA Extraction, cDNA Library Construction and Illumina Deep Sequencing

For each flower developmental stage, three individual flowers from individual plants were randomly chosen and total RNA samples were extracted from each flower by using an RNAprep pure Plant RNA Purification Kit (Tiangen Biotech, Beijing, China). The quality and quantity of total RNA were analyzed using an UltrasecTM 2100 pro UV/Visible Spectrophotometer (Amersham Biosciences, Uppsala, Sweden) and by gel electrophoresis. For each developmental stage, the RNA samples from the three individuals were then pooled together in equal amounts to generate one mixed sample. These three mixed RNA samples (*i.e.* one for each flower stage) were subsequently used in cDNA library construction and Illumina deep sequencing.

Three cDNA libraries were prepared using a TruSeq™ RNA sample preparation Kit from Illumina (San Diego, CA, USA), and using in each case 5 µg of total RNA. Messenger RNA was isolated by polyA selection with oligo (dT) beads and fragmented using fragmentation buffer; cDNA synthesis, end repair, A-base addition and ligation of the Illumina-indexed adaptors were then all performed according to the Illumina protocol. Libraries were then size-selected on 2% Low Range Ultra Agarose for cDNA target fragments of 300–500 bp; this was followed by PCR amplification using Phusion DNA polymerase (NEB) for 15 PCR cycles. Following quantification by TBS380, paired-end libraries were sequenced using Illumina HiSeq™ 2000 system (2×100 bp read length) at Shanghai Majorbio Bio-pharm Biotechnology Co., Ltd. (Shanghai, China). All raw-sequence reads data were deposited in NCBI Sequence Read Archive (SRA, http://www.ncbi.nlm.nih.gov/Traces/sra) with accession number SRA106143.

### Transcriptome *De novo* Assembly

The raw reads were cleaned by removing low-quality reads (Q value <25), adapter sequences, reads with ambiguous bases ‘N’, and fragments of less than 20 bp in length, using SeqPrep (https://github.com/jstjohn/SeqPrep) and ConDeTri_v2.0.pl software(http://code.google.com/p/condetri/downloads/detail?name=condetri_v2.0.pl). *De novo* assembly of the *Chimonanthus praecox* transcriptome in the absence of a reference genome, using the three libraries obtained from the different flower stages, was accomplished using Trinity *de novo* transcriptome assembly software [21].

### Sequence Annotation and Classification

For annotation, the sequences were searched against the NCBI non-redundant (NR) protein database [63] using BlastX, with a cut-off E-value of 10^−5^. Gene ontology (GO) terms were extracted from the annotation of high-score BLAST matches in the NCBI NR proteins database (E value ≤1.0×10^−5^) using blast2go (http://www.blast2go.com/b2ghome), and then sorted for the GO categories using in-house perl scripts [64]. Functional annotation of the proteome was carried out by a BlastP homology search against the NCBI Clusters of Orthologous Groups (COG) database (http://www.ncbi.nlm.nih.gov/COG/). KEGG pathway annotations were performed using Blastall software against the KEGG database.

### Expression Analysis

After assembling the *Chimonanthus praecox* transcriptome, every RNA-seq library was separately aligned to the generated transcriptome assembly, using Bowtie [65]. The counting of alignments was performed using the RSEM package [66]. The DEGs were analyzed using the R Bioconductor package, edgeR [67]. The P-value set the threshold for the differential gene expression test. The threshold of the P-value in multiple tests was determined by the value for the false discovery rate (FDR) [68]. We used “FDR≤0.05 and the absolute value of Log_2_ fold change (Log_2_ FC)≥2” as the threshold to judge the significance of gene expression differences. For pathway enrichment analysis, DEGs were mapped to the terms in the KEGG database by using the KOBAS program (http://kobas.cbi.pku.edu.cn/home.do). Pathways were defined as significantly enriched pathways with respect to DEGs by taking a corrected P-value ≤0.05 as the threshold.

### Real-time Quantitative PCR Validation of RNA-Seq Data

Ten DEGs involved in plant hormone transduction pathways were chosen for validation using Real-time qPCR. The primers, designed with the software Primer Primer 5.0, are listed in Table S7. Total RNA was extracted from flowers at each stage using an RNAprep pure Plant RNA Purification Kit (Tiangen Biotech, Beijing, China) and reverse transcribed into cDNA using a PrimeScript RT Reagent Kit with gDNA Eraser (TaKaRa, Otsu, Japan). The real-time polymerase chain reaction (PCR) was performed on 10 µL reactions using 5 µL of Ssofast EvaGreen Supermix (Bio-Rad, Hercules, CA), 0.5 µL of each primer (final concentration of 500 nM), 3.5 µL of water, and 0.5 µL of cDNA template. PCR was carried out using the Bio-Rad CFX96 RealTime PCR system (Bio-Rad, US), using a denaturation temperature of 95°C for 30 s, followed by 40 cycles of 95°C for 5 s and 59°C for 5 s. To control the specificity of the annealing of the oligonucleotides, dissociation kinetics were performed using the real-time PCR system at the end of the experiment (60 to 95°C, continuous fluorescence measurement). A comparative Ct method (2^–ΔΔCt^) of relative quantification [69] was used to analyze the real-time quantitative PCR data, using Bio-Rad CFX Manager Software, Version 1.6. The genes for actin and tubulin were used for the calculation of relative transcript abundance [14]. The sizes of the amplified products were confirmed through gel electrophoresis. Negative controls without templates were carried out concurrently. All quantitative PCRs for each gene used three biological replicates, with three technical replicates per experiment.

## Supporting Information

Figure S1 **The distribution of wintersweet genes in the plant hormone signal transduction pathway based on KEGG database.** The genes involved in the pathway are color-coded: green boxes, genes identified in our data; and light blue boxes, genes involved in the pathway present in KEGG database but undetectable in our data.
(TIF)

Table S1 **Sequence length distribution of transcripts assembled from Illumina reads.**
(XLS)

Table S2 **Summary of GO term assignment for the wintersweet flower transcriptome.**
(XLS)

Table S3 **Summary of COG functional classification for the wintersweet flower transcriptome.**
(XLS)

Table S4 **Summary of KEGG pathways involved in the wintersweet flower transcriptome.**
(XLS)

Table S5 **Description of the 20 most up- and down-regulated genes in DP vs OF.**
(XLS)

Table S6 **Description of the 20 most up- and down-regulated genes in OF vs SF.**
(XLS)

Table S7 **The primers used for analysis of gene expression by qRT-PCR.**
(XLS)

## References

[1] Li Y, Li PT (2000) Origin, evolution and distribution of the Calycanthaceae. Guihaia 20: 295–300.

[2] Zhang RH, Liu HE (1998) Wax Shrubs in World (Calycanthaceae). Beijing: China Science and Technology Press.

[3] Zhao KG, Zhou MQ, Chen LQ, Zhang DL, Gituru WR (2007) Genetic diversity and discrimination of *Chimonanthus praecox* (L.) Link germplasm using ISSR and RAPD markers. Hortscience 42 (5): 1144–1148.

[4] Zhao Y, Zhang Y, Wang ZZ (2010) Chemical Composition and Biological Activities of Essential Oil from Flower of *Chimonanthus praecox* (L.) Link. Lishizhen medicine and material medical research 21(3): 622–625.

[5] Deng CH, Song GX, Hu YM (2004) Rapid determination of volatile compounds emitted from *Chimonanthus praecox* flowers by HS–SPME–GC–MS. Z. Naturforsch 59: 636–640. 10.1515/znc-2004-9-1005 15540594

[6] Azuma H, Toyota M, Asakawa Y (2005) Floral scent chemistry and stamen movement of *Chimonanthu preacox* (L.) Link (Calycanthaceae). Acta Phytotax Geobot 56 (2): 197–201.

[7] Feng JQ (2007) New Zealand flower industry–with special reference to wintersweet introduction and commercialization. Journal of Beijing Forestry University (suppl 1): 4–8.

[8] Bendahmane M, Dubois A, Raymond O, Bris ML (2013) Genetics and genomics of flower initiation and development in roses. J Exp Bot 64(4): 847–857. 23364936 10.1093/jxb/ers387 PMC3594942

[9] Iordachescu M, Verlinden S (2005) Transcriptional regulation of three EIN3-like genes of carnation (*Dianthus caryophyllus* L. cv. Improved White Sim) during flower development and upon wounding, pollination, and ethylene exposure. J Exp Bot 56: 2011–2018. 15983019 10.1093/jxb/eri199

[10] Harada T, Torii Y, Morita S, Masumura T, Satoh S (2010) Differential expression of genes identified by suppression subtractive hybridization in petals of opening carnation flowers. J Exp Bot 61(9): 2345–2354. 20308205 10.1093/jxb/erq064 PMC2877890

[11] Tanase K, Nishitani C, Hirakawa H, Isobe S, Tabata S, et al (2012) Transcriptome analysis of carnation (*Dianthus caryophyllus* L.) based on next-generation sequencing technology. BMC genomics 13(1): 292. 22747974 10.1186/1471-2164-13-292 PMC3411436

[12] Davies B, Cartolano M, Schwarz-Sommer Z (2006) Flower Development: The *Antirrhinum* Perspective. Adv Bot Res 44: 279–321.

[13] Shang Y, Venail J, Mackay S, Bailey PC, Schwinn KE, et al (2011) The molecular basis for venation patterning of pigmentation and its effect on pollinator attraction in flowers of *Antirrhinum* . New Phytol 189(2): 602–615. 21039563 10.1111/j.1469-8137.2010.03498.x

[14] Sui SZ, Luo JH, Ma J, Zhu Q, Lei XH, et al. (2012) Generation and analysis of expressed sequence tags from *Chimonanthus praecox* (Wintersweet) flowers for discovering stress-responsive and floral development-related genes. Comp Funct Genomics. doi: 10.1155/2012/134596. 10.1155/2012/134596 PMC3318203 22536115

[15] Wang Z, Gerstein M, Snyder M (2009) RNA-Seq: a revolutionary tool for transcriptomics. Nat Rev Genet 10: 57–63. 19015660 10.1038/nrg2484 PMC2949280

[16] Ward JA, Ponnala L, Weber CA (2012) Strategies for transcriptome analysis in nonmodel plants. Am J B 99: 267–276. 10.3732/ajb.1100334 22301897

[17] Cloonan N, Grimmond SM (2008) Transcriptome content and dynamics at single-nucleotide resolution. Genome Biol 9: 234. 18828881 10.1186/gb-2008-9-9-234 PMC2592708

[18] Nagalakshmi U, Wang Z, Waern K, Shou C, Raha D, et al (2008) The transcriptional landscape of the yeast genome defined by RNA sequencing. Science 320: 1344–1349. 18451266 10.1126/science.1158441 PMC2951732

[19] Hoen PA, Ariyurek Y, Thygesen HH, Vreugdenhil E, Vossen RH, et al (2008) Deep sequencing-based expression analysis shows major advances in robustness, resolution and inter-lab portability over five microarray platforms. Nucleic Acids Res 36: 141. 10.1093/nar/gkn705 PMC2588528 18927111

[20] Marioni JC, Mason CE, Mane SM, Stephens M, Gilad Y (2008) RNA-seq: an assessment of technical reproducibility and comparison with gene expression arrays. Genome Res 18: 1509–1517. 18550803 10.1101/gr.079558.108 PMC2527709

[21] Grabherr MG, Haas BJ, Yassour M, Levin JZ, Thompson DA, et al (2011) Full-length transcriptome assembly from RNA-Seq data without a reference genome. Nat Biotechnol 29: 644–652. 21572440 10.1038/nbt.1883 PMC3571712

[22] Conesa A, Götz S (2008) Blast2GO: A comprehensive suite for functional analysis in plant genomics. Int J Plant Genomics 619–832. 10.1155/2008/619832 PMC2375974 18483572

[23] Mao X, Cai T, Olyarchuk JG, Wei L (2005) Automated genome annotation and pathway identification using the KEGG orthology (KO) as a controlled vocabulary. Bioinformatics 21: 3787–3793. 15817693 10.1093/bioinformatics/bti430

[24] Kitajima M, Mori I, Arai K, Kogure N, Takayama H (2006) Two new tryptamine-derived alkaloids from *Chimonanthus praecox* f. concolor. Tetrahedron Letters 47(19): 3199–3202.

[25] Van Doorn WG, Balk PA, Van Houwelingen AM, Hoeberichts FA, Hall RD, et al (2003) Gene expression during anthesis and senescence in *Iris* flowers. Plant Mol Biol 53(6): 845–863. 15082930 10.1023/B:PLAN.0000023670.61059.1d

[26] Breeze E, Wagstaff C, Harrison E, Bramke I, Rogers H, et al (2004) Gene expression patterns to define stages of post-harvest senescence in *Alstroemeria* petals. Plant Biotechnol J 2(2): 155–168. 17147607 10.1111/j.1467-7652.2004.00059.x

[27] Dubois A, Remay A, Raymond O, Balzergue S, Chauvet A, et al (2011) Genomic approach to study floral development genes in *Rosa sp* . PloS one 6(12): e28455. 22194838 10.1371/journal.pone.0028455 PMC3237435

[28] Mizutani M, Ohta D (2010) Diversification of P450 genes during land plant evolution. Annu Rev Plant Biol 61: 291–315. 20192745 10.1146/annurev-arplant-042809-112305

[29] Jones ML, Larsen PB, Woodson WR (1995) Ethylene-regulated expression of a carnation cysteine protease during flower petal senescence. Plant Mol Biol 28: 505–512. 7632919 10.1007/BF00020397

[30] Valpuesta V, Lange NE, Guerrero C, Reid MS (1995) Up regulation of a cysteine protease accompanies the ethylene insensitive senescence of daylily (*Hemerocallis*) flowers. Plant Mol Biol 28: 575–582. 7632925 10.1007/BF00020403

[31] Wagstaff C, Leverentz MK, Griffiths G, Thomas B, Chanasut U, et al (2002) Cysteine protease gene expression and proteolytic activity during senescence of *Alstroemeria* petals. J Exp Bot 53: 1–8. 11807127 10.1093/jexbot/53.367.233

[32] Eason JR, Ryan DJ, Pinkney TT, O’Donoghue EM (2002) Programmed cell death during flower senescence: isolation and characterization of cysteine proteases from *Sandersonia aurantiaca* . Funct Plant Biol 29: 1055–1064. 10.1071/PP01174 32689556

[33] Hunter DA, Steele BC, Reid MS (2002) Identification of genes associated with perianth senescence in daffodil (*Narcissus pseudonarcissus* L. ‘Dutch Master’). Plant Science 163: 13–21.

[34] Arora A, Singh VP (2004) Cysteine protease gene expression and proteolytic activity during floral development and senescence in ethylene-insensitive *Gladiolus grandiflora* . J Plant Biochem Biotech 13: 123–126.

[35] Davies PJ (2010) The plant hormones: their nature, occurrence, and functions. In: Plant hormones, Springer Netherlands, pp1–15.

[36] Depuydt S, Hardtke CS (2011) Hormone signalling crosstalk in plant growth regulation. Current Biology 21(9): R365–R373. 21549959 10.1016/j.cub.2011.03.013

[37] Shahri W, Tahir I (2011) Flower senescence-strategies and some associated events. The Botanical Review 77(2): 152–184.

[38] Sheng AW, Guo WM, Sun ZH (1999) Study on dynamics of endogenous hormones and parameters concerned senescence in cut wintersweet flowers. Journal of Beijing Forestry University 2: 48–53.

[39] Ma J, Li Z, Wang B, Sui SZ, Li MY (2012) Cloning of an expansin gene from *chimonanthus praecox* flowers and its expression in flowers treated with ethephon or 1-methylcyclopropene. Hortscience 47(10): 1472–1477.

[40] Alonso-Ramírez A, Rodríguez D, Reyes D, Jiménez JA, Nicolás G, et al (2009) Evidence for a role of gibberellins in salicylic acid-modulated early plant responses to abiotic stress in Arabidopsis seeds. Plant Physiol 150(3): 1335–1344. 19439570 10.1104/pp.109.139352 PMC2705047

[41] Santner A, Estelle M (2009) Recent advances and emerging trends in plant hormone signalling. Nature 459(7250): 1071–1078. 19553990 10.1038/nature08122

[42] Vlot AC, Dempsey DMA, Klessig DF (2009) Salicylic acid, a multifaceted hormone to combat disease. Annu Rev Phytopathol 47: 177–206. 19400653 10.1146/annurev.phyto.050908.135202

[43] Boyle P, Le Su E, Rochon A, Shearer HL, Murmu J, et al (2009) The BTB/POZ domain of the Arabidopsis disease resistance protein NPR1 interacts with the repression domain of TGA2 to negate its function. The Plant Cell Online 21(11): 3700–3713. 10.1105/tpc.109.069971 PMC2798319 19915088

[44] Dudareva N, Pichersky E (2000) Biochemical and molecular genetic aspects of floral scents. Plant Physiol 122: 627–633. 10712525 10.1104/pp.122.3.627 PMC1539243

[45] Pichersky E, Noel JP, Dudareva N (2006) Biosynthesis of plant volatiles: nature’s diversity and ingenuity. Science 311: 808–811. 16469917 10.1126/science.1118510 PMC2861909

[46] Dudareva N, Cseke L, Blanc VM, Pichersky E (1996) Molecular characterization and cell type-specific expression of linalool synthase gene from *Clarkia* . Plant Physiol 111: 815.

[47] Dudareva N, Cseke L, Blanc VM, Pichersky E (1996) Evolution of floral scent in *Clarkia*: novel patterns of S-linalool synthase gene expression in the *C. breweri* flower. The Plant Cell 8: 1137–1148. 8768373 10.1105/tpc.8.7.1137 PMC161191

[48] Dudareva N, D’Auria JC, Nam KH, Raguso RA, Pichersky E (1998) Acetyl-CoA: benzylalcohol acetyltransferase–an enzyme involved in floral scent production in *Clarkia breweri* . Plant J 14: 297–304. 9628024 10.1046/j.1365-313x.1998.00121.x

[49] Dudareva N, Raguso RA, Wang J, Ross JR, Pichersky E (1998) Floral scent production in *Clarkia breweri*. III. Enzymatic synthesis and emission of benzenoid esters. Plant Physiol 116: 599–604. 9489012 10.1104/pp.116.2.599 PMC35117

[50] Tholl D, Kish CM, Orlova I, Sherman D, Gershenzon J, et al (2004) Formation of monoterpenes in *Antirrhinum majus* and *Clarkia breweri* flowers involves heterodimeric geranyl diphosphate synthases. The Plant Cell 16: 977–992. 15031409 10.1105/tpc.020156 PMC412871

[51] Boatright J, Negre F, Chen F, Kish CM, Wood B, et al (2004) Understanding in vivo benzenoid metabolism in petunia petal tissue. Plant Physiol 135: 1993–2011. 15286288 10.1104/pp.104.045468 PMC520771

[52] Guterman I, Shalit M, Menda N, Piestun D, Dafny-Yelin M, et al (2002) Rose scent: genomics approach to discovering novel floral fragrance-related genes. The Plant Cell 14: 2325–2338. 12368489 10.1105/tpc.005207 PMC151220

[53] Spiller M, Berger RG, Debener T (2010) Genetic dissection of scent metabolic profiles in diploid rose populations. Theor Appl Genet 120(7): 1461–1471. 20084491 10.1007/s00122-010-1268-y

[54] Xiang L, Zhao K, Chen L (2010) Molecular cloning and expression of *Chimonanthus praecox* farnesyl pyrophosphate synthase gene and its possible involvement in the biosynthesis of floral volatile sesquiterpenoids. Plant Physiol and Biochem 48(10): 845–850. 20855217 10.1016/j.plaphy.2010.08.015

[55] Ma L, Li HF, Peng CC, Chen ZF, Long ZF (2012) Cloning of SAMT gene cDNA from *Chimonanthus praecox* and its expression in *Escherichia coli* . Agri Sci Techn 13(1): 88–93.

[56] Lee S, Chappell J (2008) Biochemical and genomic characterization of terpene synthese in *Magnolia grandiflora* . Plant Physiol 147: 1017–1033. 18467455 10.1104/pp.108.115824 PMC2442544

[57] Phillips MA, Wildung MR, Williams DC, Hyatt DC, Croteau RB (2003) cDNA isolation, functional expression, and characterization of (+)-α-pinene synthase and (–)-α-pinene synthase from loblolly pine (*Pinus taeda*): stereocontrol in pinene biosynthesis. Arch Biochem Biophys 411: 267–276. 12623076 10.1016/s0003-9861(02)00746-4

[58] Jones CG, Keeling CI, Ghisalberti EL, Barbour EL, Plummer JA, et al (2008) Isolation of cDNAs and functional characterization of two multi-product terpene synthase enzymes from sandalwood, *Santalum album* L. Arch Biochem Biophys. 477: 121–130. 10.1016/j.abb.2008.05.008 18541135

[59] Martin DM, Bohlmann J (2004) Identification of *Vitis vinifera* (–) α-terpineol synthase by in silico screening of full-length cDNA ESTs and functional characterization of recombinant terpene synthase. Phytochem 65: 1223–1229. 10.1016/j.phytochem.2004.03.018 15184006

[60] Lin C, Shen B, Xu Z, Köllner TG, Degenhardt J, et al (2008) Characterization of the monoterpene synthase gene tps26, the ortholog of a gene induced by insect herbivory in maize. Plant Physiol 146: 940–951. 18218975 10.1104/pp.107.109553 PMC2259071

[61] Fähnrich A, Krause K, Piechulla B (2011) Product Variability of the ‘Cineole Cassette’ Monoterpene Synthases of Related *Nicotiana* Species. Molecular Plant 4 (6): 965–984. 10.1093/mp/ssr021 21527560

[62] Pichersky E, Dudareva N (2007) Scent engineering: toward the goal of controlling how flowers smell. Trends Biotechnol 25: 105–110. 17234289 10.1016/j.tibtech.2007.01.002

[63] Pruitt KD, Tatusova T, Maglott DR (2007) NCBI reference sequences (RefSeq): a curated non-redundant sequence database of genomes, transcripts and proteins. Nucleic Acids Res 35: 61–65. 10.1093/nar/gkl842 PMC1716718 17130148

[64] Ashburner M, Ball CA, Blake JA, Botstein D, Butler H, et al (2000) Gene ontology: tool for the unification of biology. Nat Genet 25(1): 25–29. 10802651 10.1038/75556 PMC3037419

[65] Langmead B, Trapnell C, Pop M, Salzberg SL (2009) Ultrafast and memory-efficient alignment of short DNA sequences to the human genome. Genome Biol 10(3): R25. 19261174 10.1186/gb-2009-10-3-r25 PMC2690996

[66] Li B, Dewey CN (2011) RSEM: accurate transcript quantification from RNA-Seq data with or without a reference genome. BMC Bioinformatics 12: 323. 21816040 10.1186/1471-2105-12-323 PMC3163565

[67] Robinson MD, McCarthy DG, Smyth GK (2010) edgeR: a Bioconductor package for differential expression analysis of digital gene expression data. Bioinformatics 26: 139–140. 19910308 10.1093/bioinformatics/btp616 PMC2796818

[68] Benjamini Y, Yekutieli D (2001) The control of the false discovery rate in multiple testing under dependency. Ann Stat 29: 1165–1188.

[69] Livak KJ, Schmittgen TD (2001) Analysis of relative gene expression data using real-time quantitative PCR and the 2^–ΔΔCt^ method. Methods 25: 402–408. 11846609 10.1006/meth.2001.1262

