# Supplementary figures and images for: Transcriptomic Analysis of Flower Development in Wintersweet (Chimonanthus praecox)
Source: PLoS One. 2014 Jan 29;9(1):e86976. doi: 10.1371/journal.pone.0086976 (PMC3906103; doi:10.1371/journal.pone.0086976)

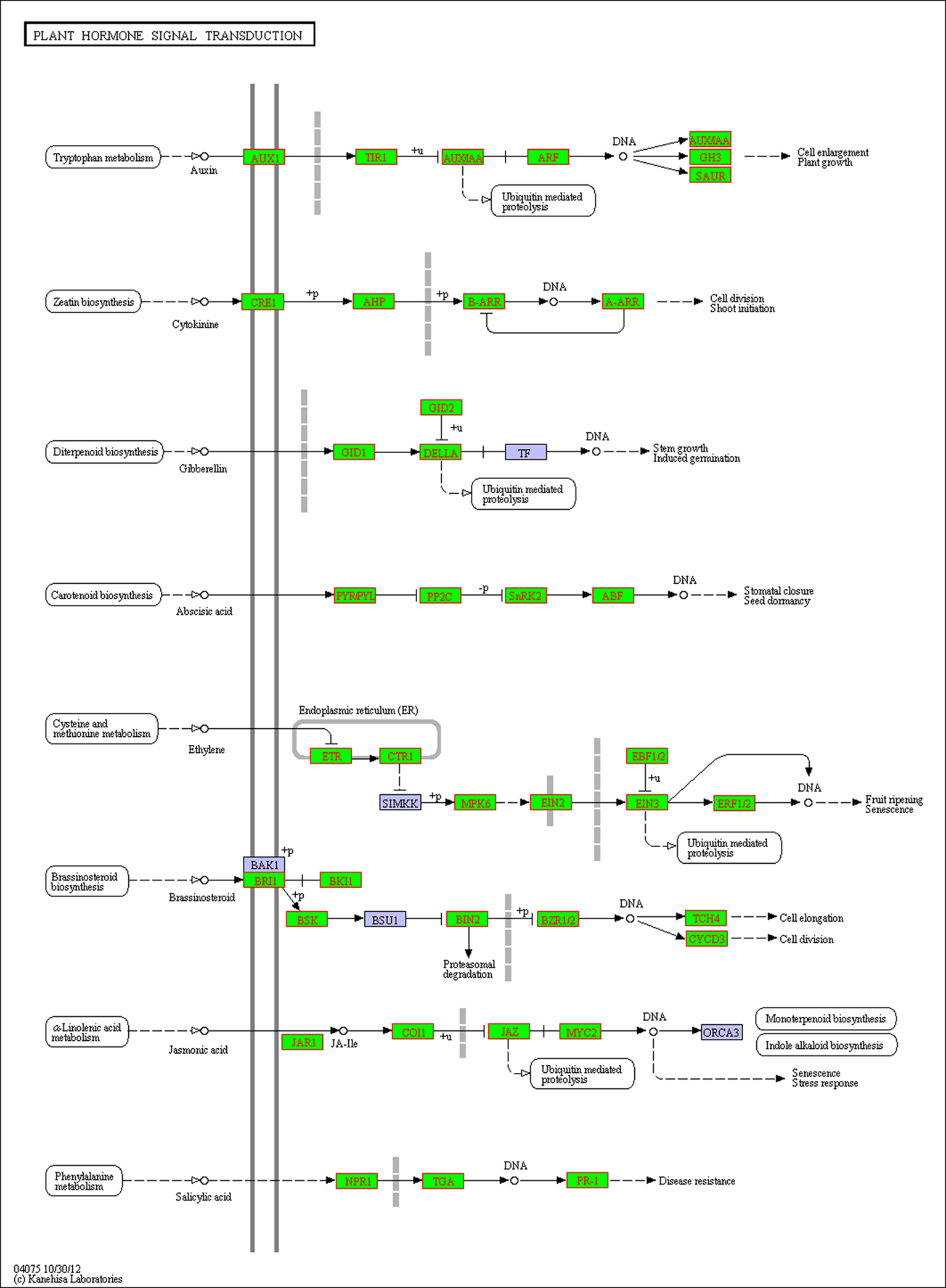

Supplement: Figure S1 — The distribution of wintersweet genes in the plant hormone signal transduction pathway based on KEGG database. The genes involved in the pathway are color-coded: green boxes, genes identified in our data; and light blue boxes, genes involved in the pathway present in KEGG database but undetectable in our data. (TIF) [file pone.0086976.s001.tif]
